# Enhanced Arsenic(V) Removal on an Iron-Based Sorbent Modified by Lanthanum(III)

**DOI:** 10.3390/ma13112553

**Published:** 2020-06-03

**Authors:** Sebastian Dudek, Dorota Kołodyńska

**Affiliations:** Department of Inorganic Chemistry, Institute of Chemical Sciences, Faculty of Chemistry, Maria Curie-Skłodowska University, pl. Marii Curie-Skłodowskiej 2, 20-031 Lublin, Poland; d.kolodynska@poczta.umcs.lublin.pl

**Keywords:** arsenic removal, lanthanum, iron oxides, adsorption, Arsen X^np^

## Abstract

Modification of a commercial iron oxide ion exchanger (Arsen X^np^) was carried out to enhance the removal of arsenic(V) ions. The modification consisted of the adsorption of lanthanum(III) ions on the Arsen X^np^ surface. After adsorption, the material was dried at 313 K to obtain the modified ion exchanger Arsen X^np^-La(III). The modification process itself was tested for optimal pH, kinetics, and equilibrium adsorption isotherm study. Accurate sorbent characteristics were made using, among others, SEM, FTIR, and nitrogen adsorption/desorption isotherms. Then, various tests were carried out to compare the adsorption properties of the modified and unmodified material. It turned out that the tested material was able to completely remove arsenic from an aqueous solution with an initial concentration of up to 50 mg/dm^3^. Without modification, it was not possible to reach the WHO recommended 10 μg/dm^3^ arsenic limit even at an initial concentration of 25 mg/dm^3^. Moreover, the maximum sorption capacity increased from 22.37 to 61.97 mg/g after modification (3 times greater than before modification). It is worth noting that the process of removing arsenic on Arsen X^np^-La(III) is fast—equilibrium is reached after about 120 min. Under almost neutral conditions, precipitation and adsorption can be the main mechanisms of As(V) removal. After modification, the removal capacity was enhanced by the co-precipitation and adsorption by exchange of the OH– group with arsenic ions. Such La(III) based adsorbent can be successfully applied in wastewater purification and displays superior performance for removing arsenic.

## 1. Introduction

Arsenic is an element known for its toxicity. It is found in South and North America, Europe, Australia and Africa, but the largest problem occurs in the areas of southern and south-east Asia where the maximum permissible level of arsenic is largely exceeded [1,2]. Arsenic penetrates into water systems as a result of natural weathering processes, volcanic emissions, geochemical reactions or anthropogenic activities, e.g., fossil fuel combustion, the use of arsenic pesticides and herbicides, and mining activities [3,4].

According to the World Health Organization (WHO) guidelines, the admissible content of arsenic in drinking water is 10 μg/dm^3^. Therefore, more than 100 million people around the world are at risk of its exposure. However, 45 million people from developing Asian countries are still exposed to arsenic concentration greater than 50 μg/dm^3^. Long-term consumption of arsenic contaminated water can lead to neurological disorders, skin pigment changes, vomiting and also kidney, lung or bladder cancer [5]. Therefore high-efficiency and cost-effective technologies have been needed to treat and remove arsenic from drinking water.

In numerous available studies, adsorption has been chosen as the most appropriate method for solving the problem of water systems contaminated by arsenic(V). Adsorbents which combine the best advantages, i.e., low cost, great strength, and the ability to be used in changing environmental conditions, are desirable. Many authors decide to exploit ion exchangers based on iron oxides [6,7,8]. However, these adsorbents often have low adsorption capacity towards arsenic ions. Consequently, a lot of authors try to modify them in order to obtain greater adsorption. Moreover, to enhance sorption capacity and selectivity, immobilization of ligands with multi-coordinating active sites, ligands which are biomimetically relevant, complexes with low molecular weight and immobilizing of ethers, calixarenes, impregnation of metal ions, and preparation of reactive ion exchangers are proposed. In the paper by Jiang et al. [9], adsorption of arsenic(V) onto polystyrene-iron oxide PS-Fe_3_O_4_ sorbent was tested depending on the pH of the solution, arsenic concentration, and phase contact time. Additionally, the effect of accompanying anions on the efficiency of the process was checked. PS-Fe_3_O_4_ was able to reach equilibrium faster and exhibited higher sorption capacity for arsenates(V) than the unmodified Fe_3_O_4_ (139.3 mg/g—77.7% higher than pure Fe_3_O_4_). Chloride and nitrate(V) anions, unlike PO_4_^3−^ and SiO_3_^2−^ ions, did not have a major impact on the sorption process. The discussed hybrid material was effectively separated from the water in the presence of a weak magnetic field (<0.035 T). In addition, it could be reused after regenerating the sorbent with NaOH solution.

This type of adsorbent containing inbuilt iron nanooxide molecules of the goethite structure—Purolite Arsen X^np^—is produced on a commercial scale. In the production process, iron oxide was distributed in the pores of the above-mentioned ion exchanger as a layer of a few nanometers thickness. However, in order to increase the surface effectiveness of such adsorbents, other modification methods can be applied. One of them could be modification of the R-N(CH_3_)_3_(OH)/FeO(OH) structure by trivalent or tetravalent ions. In the study by He et al. [10], an iron-cerium bimetal oxide adsorbent was applied for As(V) removal sorption capacity (149.84 mg/g) more successfully than many reported adsorbents. The bimetal oxide adsorbents were prepared by a co- precipitation method. Based on the XPS results, it was proved that the obtained sorbent was composed of hydroxyl (30.8%) and CeO_2_ and Fe_3_O_4_ (12.6% and 19.6%). Therefore, both the integral area of the As-O band and the As(V) adsorption capacity increased almost linearly with the decrease in the integral area of M-OH bands, proving that the adsorption of As(V) is mainly realized through the mechanism of quantitative ligand exchange. Analogous results were obtained using Fourier transform infrared spectra where it was revealed that in As(V) adsorption M-OH groups play an important role.

In this study, attempts have been made to enhance the removal of arsenate ions onto Arsen X^np^ modified by La(III) in order to reduce their amount in the environment. The sorbent was prepared by adsorption of lanthanum(III) ions under specific conditions and then for arsenic(V) removal. Lanthanum is one of the cheapest rare-earth elements. Comparison of the sorption properties of the raw Arsen X^np^ and the modified sorbent Arsen X^np^–La(III) was made. There are studies in which various sorbents have been modified with lanthanum. Jang et al. [11] synthesized the highly ordered mesoporous silica, SBA-15, and incorporated various amounts of lanthanum oxide into it. Then, they applied the material for arsenate removal in the adsorption process. Tan et al. [12] fabricated a novel adsorbent CSN-La of lanthanum immobilized on electrospun chitosan nanofiber (CSN). The saturated adsorption capacity of CSN-La reached up to 83.6 mg As(V)/g, which was significantly higher than that of CSN. The exhausted CSN-La could be repeatedly used after being eluted by sodium hydroxide solution. In the paper by Lingamdinne et al. [13], the surface of graphene oxide was functionalized with lanthanum to produce porous flowered graphene oxide-lanthanumfluoride (GO-LaF) nanocomposite. The adsorption results obtained under different conditions suggest that As(V) adsorption onto GO-LaF occurs through mixed processes such as electrostatic, ion-exchange, and surface complexation. However, a study, in which a commercially available sorbent based on iron oxide was modified with lanthanum, does not exist. A successful modification can help supply safe arsenic-free drinking water. Moreover, the multiple use of the same material can reduce significantly the costs of the groundwater treatment process.

## 2. Materials and Methods 

### 2.1. Modification of the Adsorbent

In order to increase As(V) sorption capacity, the modification of the sorbent consisted in adsorption of lanthanum(III) ions from aqueous solution (t = 6 h, c_o_ = 100 mg/dm^3^) and then drying the sorbent at 313 K. In that way, Arsen X^np^ modified with La(III) ions was obtained. To learn more about the sorbent modification process, the effects of pH and sorption kinetics of lanthanum were studied. Moreover, Langmuir and Freundlich isotherms were determined for this process. The abbreviation X^np^ will be used for the pure Arsen X^np^ sorbent, while X^np^-La(III) for Arsen X^np^ with adsorbed lanthanum(III) ions.

### 2.2. Materials and Chemicals

Purolite Arsen X^np^ (Purolite, Philadelphia, PA, USA) is a macroporous, selective resin based on polystyrene crosslinked with divinylbenzene skeleton with sulfonic groups and a unique structure of hydrated iron nanoparticles (designed to remove arsenic(V) and arsenic(III) ions). This ion exchanger is characterized by high durability and selectivity towards Cu(II), Zn(II), Cr(VI), Cd(II), and Pb(II) ions [14,15]. It is available on a commercial scale due to the finding of SenGupta and Cumbal (U.S. Patent US7291578B2 [16]) and used to impregnate strongly basic anion exchange resins with iron hydroxide nanoparticles. Next, the technology was further modified and commercialized by SolmeteX, Layne Christensen Company (The Woodlands, TX, USA), and Purolite International Ltd. Its physicochemical properties are collected in Table 1.

The point of zero charge (pH_pzc_) before and after the lanthanum(III) sorption of the sorbents was determined using the drift method [17]. The determination of pH_pzc_ was conducted by adjusting the pH of 20 mL 0.01 M NaCl solution to a value between 2 and 11 (pH_i_). 0.1 g of the sorbent was added and the final pH (pH_f_) was measured after 6 h under agitation. The pH_pzc_ is the point where pH_i_–pH_f_ = 0. The initial and final pHs were measured using the Radiometer PHM 84 pH meter (Copenhagen, Denmark) with the glass REF 451 and calomel pHG 201-8 electrodes. 

The morphology of the sorbent before and after the lanthanum(III) sorption was determined using scanning electron microscopy (SEM) (Tescan, Brno, Czech Republic) with an extended depth of field (EDF) function. 

Using an ASAP 2405 analyzer (Micromeritics, Norcross, GA, USA), porous structure parameters of the sorbents were evaluated. These tests were aimed at determining nitrogen adsorption-desorption isotherms of the analyzed materials. The specific surface area was determined by the Brunauer-Emmett-Teller method as well as the total volume and pore size distribution according to the Barret-Joyner-Halenda model. 

Infrared spectroscopy analysis with Fourier transformation was used to record the spectra of the analyzed adsorbents. X^np^ and X^np^-La(III) before and after the As(V) sorption were tested by the attenuated total reflection method using the Agilent Cary 630 FTIR spectrometer. Thanks to this, it was possible to define the characteristic functional groups located on the surface of the material used. In the FTIR analyses, infrared radiation covered the wavenumber range of 4000–650 cm^−1^.

The solutions in the model wastewater were prepared for experiments by dissolving La(NO_3_)_3_∙6H_2_O and Na_2_HAsO_4_∙7H_2_O in water. The specific pH of the solutions was achieved by adding appropriate amounts of 1 M hydrochloric acid and 1 M sodium hydroxide. All chemicals applied were used at analytical purity and purchased from POCh (Gliwice, Poland).

### 2.3. Batch Experiments

#### 2.3.1. Sorbent Modification

To examine lanthanum(III) adsorption depending on pH values, solutions containing 10 mg/dm^3^ of lanthanum(III) at pH in the range from 2 to 6 were prepared. 0.1 g of X^np^ and 20 cm^3^ of lanthanum(III) solution at different pH values were added separately to 100 cm^3^ conical flasks. After that, the samples were shaken for 24 h (c = 10 mg/dm^3^, pH = 2–6, shaking speed 180 rpm, temperature 295 K). The ELPIN+ type 358A shaker (Lubawa, Poland) was used. After shaking, the solutions were separated from the sorbent by filtration on filter paper. After the experiment, the lanthanum(III) concentrations of the analyzed ions were determined using inductively coupled plasma optical emission spectrometry (ICP-OES) (720 ES, Varian, Palo Alto, CA, USA). The wavelength used for the analysis of La(III) was 333.749 nm. The ICP-OES instrument was calibrated using the appropriate standards. To prepare all standards and blank samples, ultrapure nitric acid was used in order to avoid any matrix interference.

The sorption kinetics of lanthanum(III) was investigated using the static method. The solutions of lanthanum(III) were prepared at concentrations of 10, 50, and 100 mg/dm^3^ (and at the previously determined optimal pH value). 0.1 g of X^np^ was added to Erlenmeyer flasks. 20 cm^3^ of the above-mentioned solutions was added to each flask and shaken for 1, 3, 5, 7, 10, 20, 30, 60, 120, 240, and 360 min. After shaking, the samples were filtered on filter paper and the solutions were separated from the sorbent. Then, the concentrations of lanthanum(III) were determined using ICP-OES.

The amount of adsorbed lanthanum(III) ions (*q_t_*) was estimated from the following equation:

(1) $$q_{t}=\left(c_{0}-c_{t}\right)\times \frac{V}{m}$$

where: *q_t_* is the amount of lanthanum(III) adsorbed at time *t* (mg/g), *c*_0_ is the initial concentration of lanthanum(III) in the solution (mg/dm^3^), *c_t_* is the concentration of lanthanum(III) in the solution after time *t* (mg/dm^3^), *V* is the volume of the solution containing lanthanum(III) ions (dm^3^), *m* is the mass of sorbent (g).

The percentage of adsorption (%*S*) is that of lanthanum(III) adsorbed on the adsorbent beads calculated from the following equation:

(2) $$\%S=\frac{\left(c_{0}-c_{t}\right)}{c_{0}}\times 100\%$$

The kinetic parameters of metal ions sorption onto the sorbent were determined using the pseudo-first order kinetic equation (PFO) and pseudo-second order equation (PSO) [18,19,20]:

(3) $$\log(q_{e}-q_{t})=\log(q_{e})-\frac{k_{1}}{2.303}\times t$$

(4) $$\frac{t}{q_{t}}=\frac{1}{k_{2}\times q_{e}^{2}}+\frac{t}{q_{e}}$$

where: *q_e_* is the amount of lanthanum(III) adsorbed at equilib¬rium (mg/g), *q_t_* is the amount of lanthanum(III) adsorbed at time *t* (mg/g), *k*_1_ and *k*_2_ are the reaction rate constants of the pseudo-first order (1/min) and pseudo-second order (g/mg min).

The adsorption capacity of X^np^ towards lanthanum(III) ions was determined by the equilibrium adsorption isotherm study. The initial concentrations of La(III) ions were equal to 10, 50, 100, 150, 300 and 500 mg/dm^3^ (t = 6 h, shaking speed 180 rpm, temperature 295 K).

#### 2.3.2. Influence of pH

The solutions of 25 mg/dm^3^ As(V) ions were prepared to examine arsenic(V) adsorption depending on the pH values. The same parameters were used as when testing the effect of pH on lanthanum(III) sorption, as described in Section 2.3.1. After the experiment, the concentrations of the analyzed ions and the sorption capacities were determined using the spectrophotometric method (Cary 60, Agilent Technologies) at a wavelength 870 nm by obtaining coloured As(V) complex compounds with ammonium molybdate. The UV-Vis instrument was calibrated using the appropriate standards.

#### 2.3.3. Kinetic Studies

The arsenic(V) sorption kinetics was investigated using the static method. The solutions of arsenate(V) at concentrations of 25, 50, and 100 mg/dm^3^ (pH 6). 0.1 g of X^np^ were added to Erlenmeyer flasks. 20 cm^3^ of the above-mentioned solutions was added to each flask and shaken for 1, 3, 5, 7, 10, 20, 30, 60, 120, 240 and 360 min (shaking speed 180 rpm). The experiment was repeated for the X^np^-La(III) sorbent and the results on the modified and unmodified X^np^ were compared. The laboratory water bath shaker Elpin+ type 357 (Elpin Plus, Lubawa, Poland) was used for the experiments. After shaking, the samples were filtered on filter paper and the solutions were separated from the sorbent. Then, the concentrations of arsenic(V) ions were determined using the UV-Vis method (Cary 60, Agilent Technologies, Santa Clara, CA, USA), as described below.

The amount of adsorbed arsenic ions was estimated from Equation (1). The experimental data of As(V) kinetic studies was fitted to the PFO (Equation (3) and the PSO (Equation (4)) models.

#### 2.3.4. Equilibrium Adsorption Isotherm Study

The adsorption capacity of X^np^ towards arsenic(V) ions was determined by the equilibrium adsorption isotherm study. The same study was carried out for X^np^-La(III). The initial concentrations of As(V) ions were equal to 25, 50, 100, 150, 300, 500, 750, and 1000 mg/dm^3^ (t = 6 h, shaking speed 180 rpm, temperature 295 K). The adsorption data was fitted into both the Langmuir isotherm and the Freundlich isotherm, whose equations are presented below [18,19,20]:

(5) $$q_{e}=\frac{q_{0}K_{L}c_{e}}{1+K_{L}c_{e}}$$

(6) $$q_{e}=K_{F}c_{e}^{1/n}$$

where: *q*_0_ is the maximum monolayer adsorption capacity (mg/g); *K_L_* is the Langmuir constant (dm^3^/mg); *c_e_* is the adsorbate concentration in the solution at equilibrium (mg/dm^3^); *K_F_* is the Freundlich isotherm constant (mg/g); *n* is the Freundlich exponent (degrees of sorption favourability and intensity) (-). 

The two parameters (*K_L_* and *q_e_*) can be obtained using the slope and the intercept to characterize the adsorption process. The *R_L_* parameter is determined from the equation:

(7) $$R_{L}=\frac{1}{1+K_{L}c_{0}}$$

#### 2.3.5. Desorption

Three cycles of sorption and desorption of arsenate(V) ions were carried out on 0.1 g of the modified and unmodified sorbent. Sorption was conducted from a solution with an initial arsenic concentration of 100 mg/dm^3^, volume 20 cm^3^, temperature 295 K, pH 6, and shaking time 6 h (180 rpm). The desorption conditions were identical, and the desorbing agent was 1 M NaOH. One sorption and desorption cycle were also carried out for NaOH concentrations 0.05 and 0.2 M. After each sorption and desorption, the sorbent was separated from the solution and dried at a temperature of 313 K. Then, the ion exchanger reusability was evaluated. 

## 3. Results and Discussion

### 3.1. Sorbent Characterization

**Figure 1:**
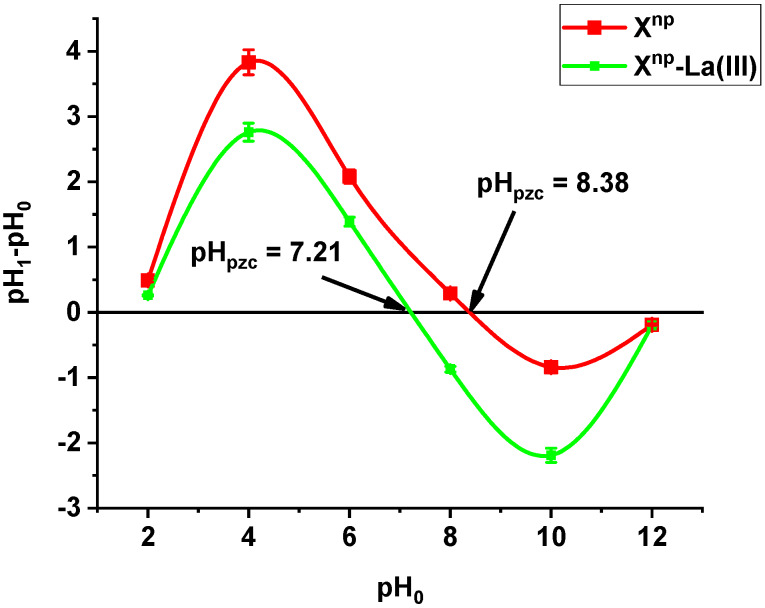
pH_pzc_ measured by the drift method for X^np^ before and after La(III) adsorption.

The point of zero charge pH_pzc_ of X^np^ is equal to 8.38. After adsorption of lanthanum (III) ions, the pH_pzc_ decreased to 7.21 (Figure 1). The pH_pzc_ value of a given substance depends on the nature of the surface [17,18]. It means that X^np^ and X^np^-La(III) are able to exchange anions at a pH below 8.38 and 7.21, respectively. Analysing the adsorption data at different pH values in connection with the speciation distribution of the As(V) forms dependent on the pH_a_ values as well as pH_ZPC_ will provide useful information about sorption mechanism. At pH > pH_ZPC_ or pH < pKa the electrostatic repulsion reduces the adsorption when pH increases (at pH > pH_ZPC_) or decrease (when pH <pKa). However, at pKa < pH < pH_ZPC_ the electrostatic attraction is an important interaction. The solution pH affected largely the behaviour of the functional groups. If the number of negative groups decreases with the decreasing solution pH, the surface becomes positively charged. Additionally, due to the high electrostatic repulsive forces involved in the adsorbent- adsorbate and adsorbate-adsorbate interactions, the adsorption capacity of X^np^-La(III) towards As(V) can be lower than when the solute is in its molecular form or if the solution pH is below pH_ZPC_. Therefore, the maximum removal of arsenic oxyanions can be observed in the acidic systems. pH_ZPC_ of X^np^-La(III) was 7.21 therefore at pH lower than this value protonation proceeds thus negatively charged arsenic species can be attracted electrostatically. As was mentioned during the discussion the mechanism of As(V) removal is more complicated. For As(V), this indicates that the attractive interaction between the positively charged surface of adsorbents and anionic As(V) species can be a major factor for arsenic removal. Similar results were obtained by Yoon et al. [19] who investigated magnetite-graphene oxide and magnetite-reduced graphene oxide composite for As(III) and As(V) removal. 

**Figure 2:**
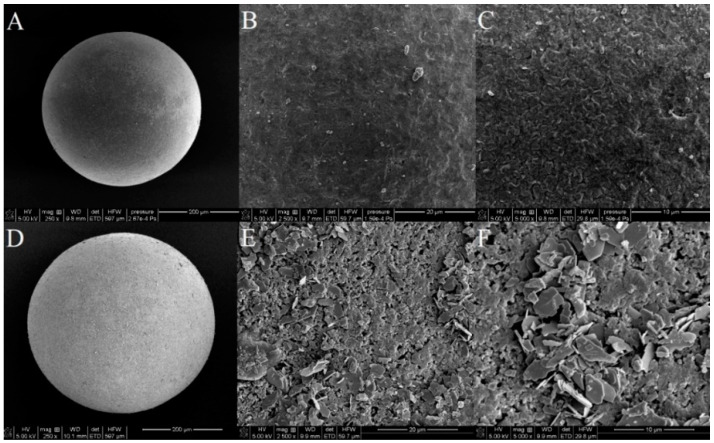
SEM images of X^np^ before (**A**–**C**) and after the modification with La(III) ions (**D**–**F**) at various magnifications (250×; 2500× and 5000×).

The SEM images of the raw and lanthanum-modified sorbent are presented in Figure 2.

The use of SEM scanning electron microscopy allowed a visual assessment of the surface structure of the tested sorbents. On the basis of the conducted tests, it can be assumed that after the sorption of lanthanum changes occur in the morphology of the sorbent surface, which becomes more heterogeneous. La (hydr)oxide nanoparticles and nanoflakes of several hundred nanometers spread over the surface. After the adsorption of As(V) process, morphology of X^np^-La(III) changes to be more “fluffy” similarly to the results presented in the paper by Vijaykumar et al. [21]. When the particle structure presents zero or low porosity, the specific surface area of the particle is a function of these attributes. Specific surface area, particle size as well as the presence of the functional groups are three of the most important parameters for sorptive materials of different types because they are related to the adsorption capacity and rate of the sorption processes. The smaller the size of the material, the higher is the surface area obtained. However, after aggregation of the particles during the La (hydr)oxide formation the surface area decreases.

**Figure 3:**
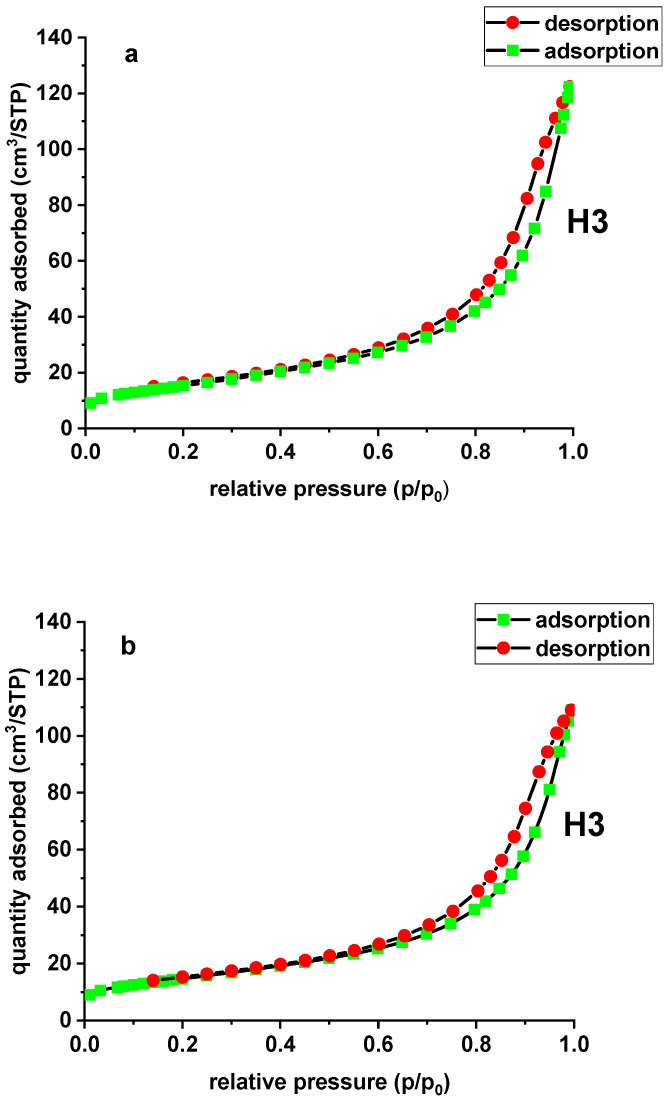
N_2_ adsorption/desorption isotherms of X^np^ (**a**) and X^np^-La(III) (**b**).

The adsorption isotherms of N_2_ on the carbon adsorbers were used to determine surface characteristics, including the BET surface area and the micropore and mesopore volumes. In the case of the modified and unmodified sorbent, the N_2_ adsorption and desorption isotherm is type IV according to the International Union of Pure and Applied Chemistry (IUPAC) classification. For type IV there appears a hysteresis loop, which is associated with capillary condensation in the pores. The sorbent is characterized by the presence of micro- and mesopores. The initial part of the isotherm is associated with single and multilayer adsorption of N_2_ because it resembles the beginning of the type II isotherm. The adsorption hystereses (type IV and V) are classified and it is widely accepted that there is a correlation between the shape of the hysteresis loop and the texture (e.g., pore geometry, pore size distribution and connectivity) of a mesoporous material. An empirical classification of hysteresis loops was given by IUPAC, which is based on an earlier classification of hysteresis by de Boer. The tested materials demonstrate H3 hysteresis due to the slit-shaped pores [22]. The hysteresis loop shapes of X^np^ and X^np^-La(III) are presented in Figure 3.

The specific surface area (*S_BET_*) was evaluated based on the BET multilayer adsorption. The total pore volume (*V_t_*) was determined from the adsorbed nitrogen volume at *p*/*p*_0_ = 0.99. The average pore diameter (*D_p_*) is estimated from the pore volume, assuming a cylindrical pore geometry and using the equation 4*V_t_*/*S_BET_*. The mesopore distribution curve was obtained from the adsorption branch of the N_2_ isotherm by the Barett–Joyner–Halenda (BJH) method. The specific surface area of X^np^ is equal to 55.27 m^2^/g and its total pore volume is 0.189 cm^3^/g. Due to lanthanum(III) adsorption and the formation of nanoflakes, X^np^-La(III) demonstrates slightly lower values of the specific surface area and total pore volume, equal to 52.75 m^2^/g and 0.168 cm^3^/g, respectively. There was also a slight difference in the average pore diameter of the unmodified sorbent (13.70 nm) and the modified one (12.79 nm). The data are presented in Table 2.

**Figure 4:**
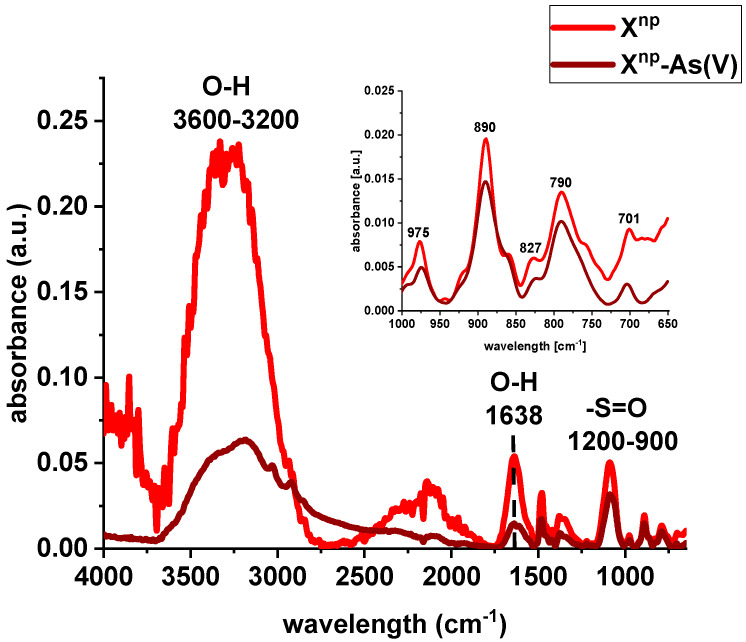
FTIR spectra of X^np^ before and after sorption of As(V) ions.

**Figure 5:**
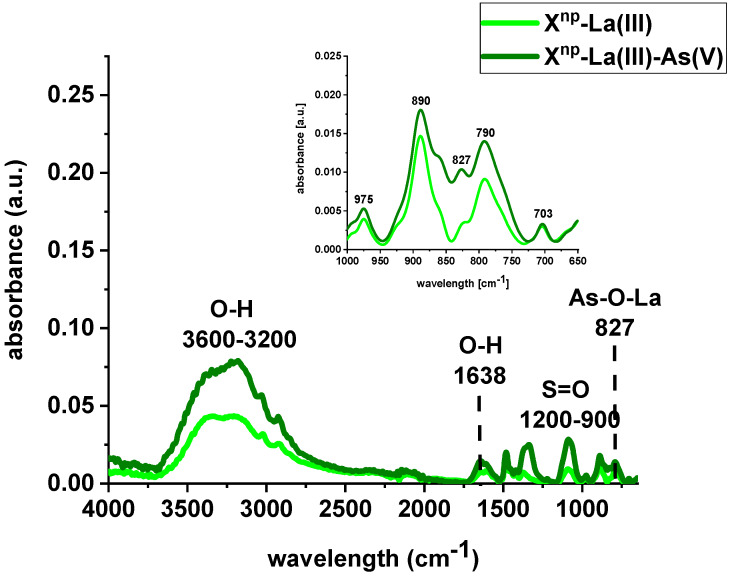
FTIR spectra of X^np^-La(III) before and after sorption of As(V) ions.

FTIR spectra of X^np^ and X^np^-La(III) were collected before and after sorption of As(V) ions (Figure 4 and Figure 5). The bands at 3600–3200 cm^−1^ indicate an O-H stretching group on the surface of iron oxide. The peak at 1638 cm^−1^ is observed to be the deformation vibration of water molecules physisorbed on the adsorbent. In comparison to the pure sorbent, the intensity of the above-mentioned peaks decreased after sorption of lanthanum and/or arsenic. Moreover, the absorption bands in the 1200–900 cm^−1^ region are attributed to the S=O stretching vibrations in the –SO_3_− groups, i.e., asymmetric and symmetric, as well as stretching of single S–OH. The increase of the peak intensity at 827 cm^−1^ was assigned to As-O-La bonding after the As(V) sorption onto X^np^-La(III). In their paper, Jais et al. demonstrated two peaks at 808 and 839 cm^−1^ derived from the As-O-La group [23]. These can indicate that arsenate(V) ions were primarily bound as a surface complex. Additionally, based on paper [24], it was found that with the increasing sorption time, the intensity of the band corresponding to the vibrations of As-O bond was progressively increased which is correlated with the fact that the amount of adsorbed As(V) also changed over the pH values. What is more, the peak position is shifted from 839 cm^−1^ to 819 cm^−1^ with the pH change from 5 to 9. In the case of the sorption of As(V) on silica derived sorbent [11] the peak around 960 cm^−1^ is connected with the La(III) modification. The Author found that in the case of the increase in lanthanum impregnation degree the absorbance intensities at 960 cm^−1^ decreased due to formation of Si–O–La bonds. It can be suggested that the monolayer phase of lanthanum oxide may be dominant at below lower lanthanum impregnation, while the multilayer phase of lanthanum oxide may be dominant at the higher lanthanum impregnation. However, the peak at 790 cm^−1^ is connected with the PS-DVB ion exchanger structure.

### 3.2. Sorbent Modification

**Figure 6:**
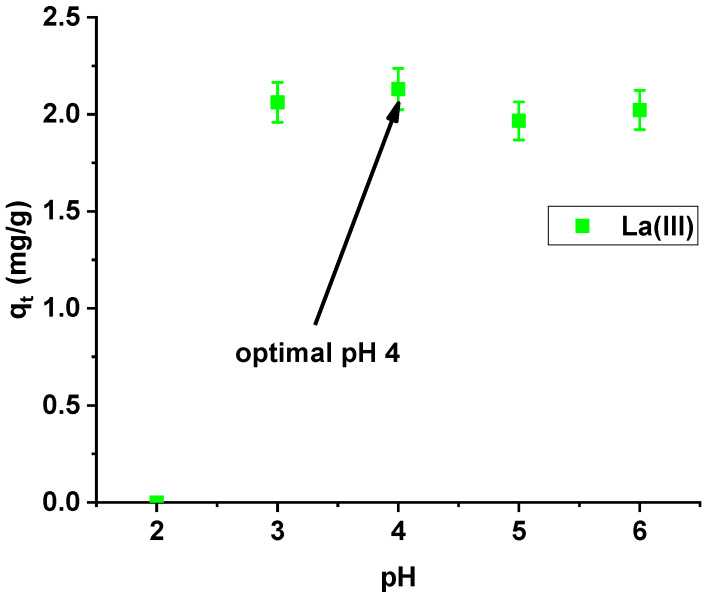
Effect of pH on lanthanum(III) adsorption on X^np^ (c_0_ = 10 mg/dm^3^; m = 0.1 g; t = 24 h).

Studying the effect of pH on La(III) sorption on X^np^, it was found that the maximum sorption capacity was obtained at pH 4 (Figure 6). At pH > 6, the precipitation of La(OH)_3_ occurred and therefore lanthanum(III) adsorption became impossible to proceed. 

Sorption capacities at the pH range from 3 to 6 are similar, but slightly higher at pH 4, so this value was selected for further studies.

**Figure 7:**
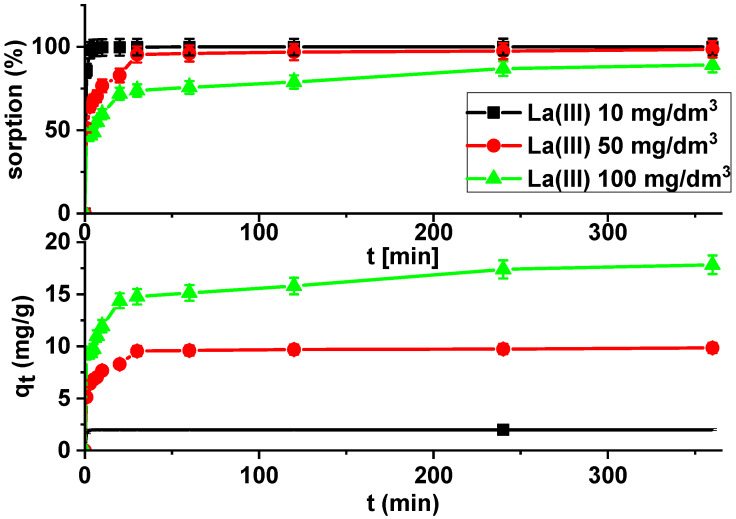
Lanthanum(III) sorption efficiency and sorption capacities as a function of time on X^np^ (c_0_ = 10, 50 and 100 mg/dm^3^, pH = 4, shaking speed 180 rpm, temperature 295 K).

Kinetic studies show that the La(III) adsorption processes are quite fast. It is observed that sorption efficiency is greater for solutions with lower initial concentrations (Figure 7). 

**Figure 8:**
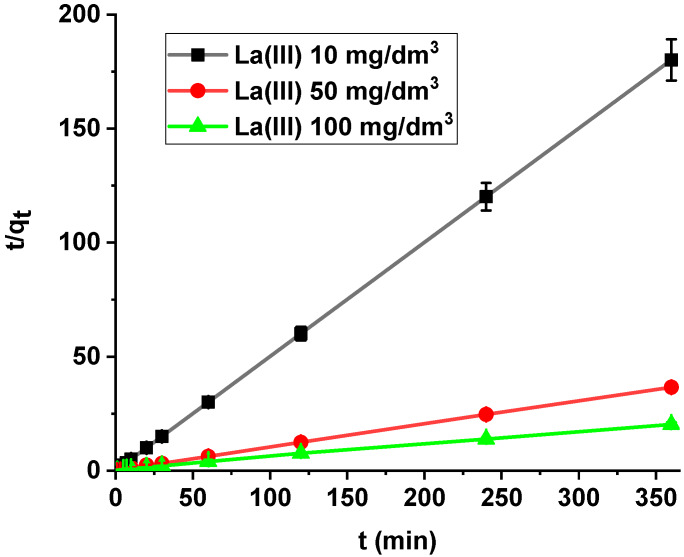
PSO plots of lanthanum(III) adsorption on X^np^ (c_0_ = 10, 50, 100 mg/dm^3^, pH = 4, shaking speed 180 rpm, temperature 295 K).

It was found that the linear regression coefficient based on the pseudo-second order (PSO) model (*R*^2^ > 0.99) gave the best fitting (Table 3, Figure 8). Based on the PSO model, the calculated adsorption capacities (1.99, 9.84, 17.82) were nearly the same as the experimental ones. The adsorption rate constants decreased with the increase in La(III) initial concentration. 

**Figure 9:**
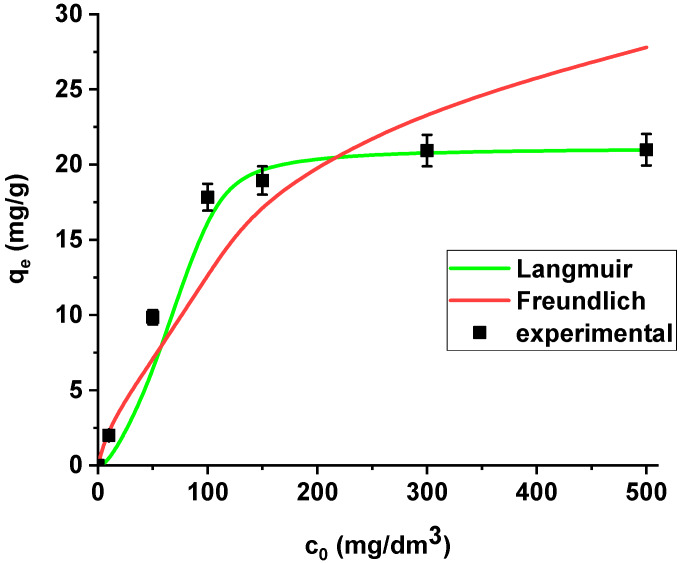
The Langmuir and Freundlich sorption isotherms of La(III) on X^np^ (pH 4, shaking speed 180 rpm, temperature 295 K).

The isotherm data were analysed using the Langmuir and Freundlich models (Figure 9).

La(III) adsorption was described better by the Langmuir model (*R*^2^ = 0.9998) than the Freundlich model (*R*^2^ = 0.8996). X^np^ demonstrates the maximum sorption capacity towards lanthanum(III) equal to 21.08 mg/g. It is worth mentioning that the coefficient *R_L_* lies in the range 0-1 (0.171), which indicates that the process is favourable.

### 3.3. Influence of pH on As(V) Adsorption on X^np^-La(III) 

Arsenic is present in compounds at various oxidation states, but the most common ones are −3, 0, +3, and +5 [25]. Arsenic(V) is more readily adsorbed on solid surfaces than arsenic(III), so in order to remove arsenic effectively, all forms of arsenic should be oxidized to the +5 oxidation state. Moreover, inorganic arsenic compounds are more toxic than organic ones, and as for the oxidation state, arsenic(III) is usually more toxic than arsenic(V). The most common inorganic forms of arsenic are arsenic(III) and arsenic(V), the amount of which depends on the pH and redox potential. At pH 3–9, arsenic(III) dominates as undissociated H_3_AsO_3_, while arsenic(V) exists as the anions HAsO_4_^2−^ and H_2_AsO_4_^−^ (pK_1_ =2.2, pK_2_ =7.1, pK_3_ =11.5) [26]. At pH 6, arsenic(V) exists as the anions HAsO_4_^2−^ and H_2_AsO_4_^−^. 

**Figure 10:**
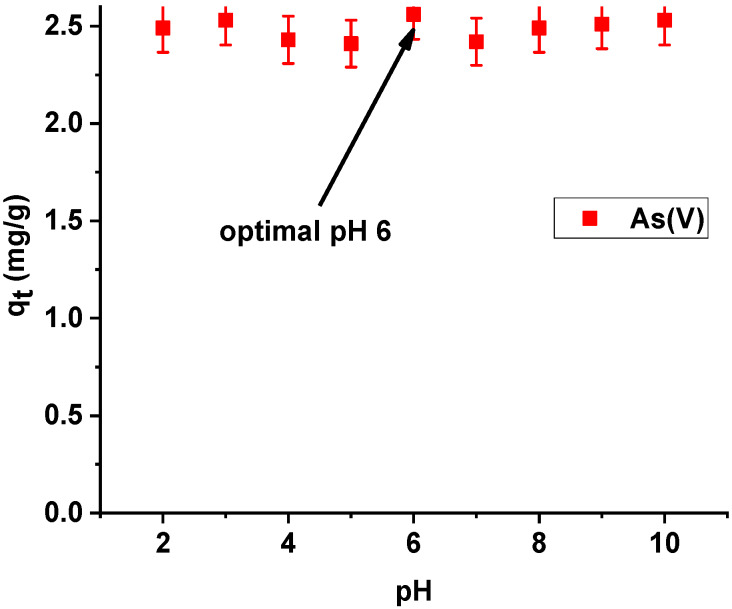
Effect of pH on adsorption of arsenic(V) on X^np^-La(III) (c_0_ = 10 mg/L; m = 0.1 g; t = 24 h, T = 295 K, shaking speed 180 rpm).

On the basis of the presented results of dependence of sorption capacities of the tested hybrid material on the initial pH of the arsenic(V) solution, the sorption capacities of X^np^-La(III) were observed to be almost equal. The highest efficiency of arsenic(V) sorption was achieved at pH 6 (Figure 10). At pH 6, arsenic(V) exists as the anions HAsO_4_^−^ and H_2_AsO_4_^−^. As follows from the speciation distribution the ratio of HAsO_4_^2−^ and H_2_AsO_4_^−^ are about 10%: 90% [27]. Consequently, they can be more readily adsorbed on an electrically charged surface. Additionally, as follows form the paper by Sarkar et al. [28] it is worth noting that the chronic toxicity caused by the presence of low concentration of arsenic (well below 1 mg/L) is not influenced by the relative distribution of As(III) and As(V). At such low concentration, As(V) gets instantaneously converted to As(III). That is why the World Health Organization (WHO), the United States Environmental Protection Agency (USEPA), and the European Union (EU), specify only the total amount of arsenic for the maximum contamination level (MCL) in drinking water.

Thus, the pH value of 6, which is slightly lower than the pH of municipal water, was selected for further studies for both X^np^ and X^np^-La(III) to ensure uniform conditions of arsenic (V) ions. In addition, a pH of 6 is a value below the pH_pzc_ of X^np^ and X^np^-La(III). The positively charged surfaces of the modified and unmodified sorbent should attract hydrogen arsenate(V) and dihydrogen arsenate(V) anions.

### 3.4. Kinetic Studies of As(V) on X^np^-La(III)

**Figure 11:**
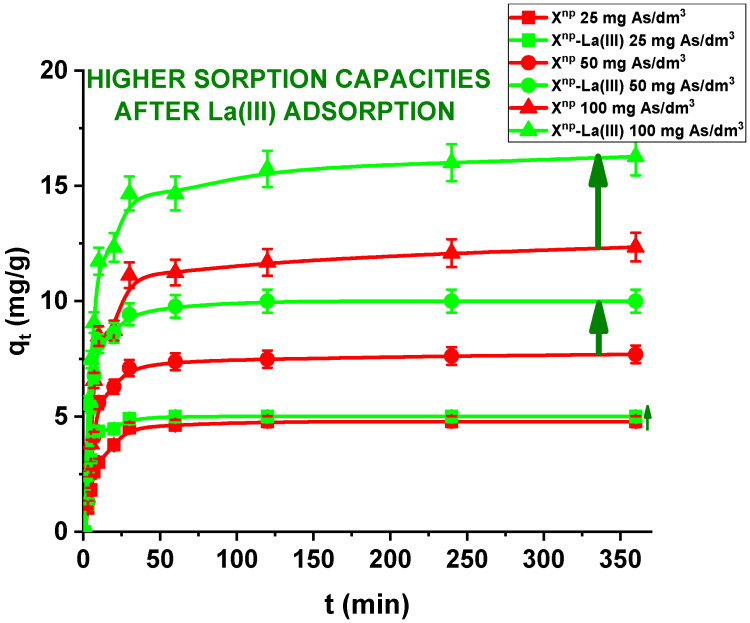
Sorption of arsenic(V) on X^np^ and X^np^-La(III) as a function of time (c_0_ = 25, 50 and 100 mg/dm^3^, pH = 6, shaking speed 180 rpm, temperature 295 K).

**Figure 12:**
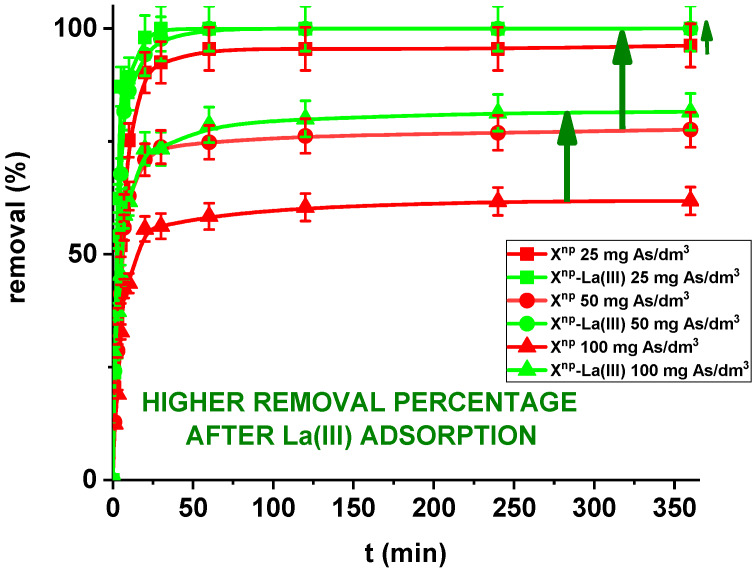
Arsenic(V) sorption efficiency for X^np^ and X^np^-La(III) (c_0_ = 25, 50 and 100 mg/dm^3^, pH = 6, shaking speed 180 rpm, temperature 295 K).

In the case of a static method, for all practical purposes the choice of experimental variables usually narrows down to two: the concentration and amount of sorbent. In the presented results, for comparison the adsorption studies were carried out for X^np^ before and after modification. Figure 11 and Figure 12 show that all the presented adsorption processes are quite fast. It is observed that sorption efficiency (%) is greater for the solutions with lower initial concentrations. For example, in the case of X^np^ at the highest adsorption percentage the concentration of arsenic(V) was reduced from 25 mg/dm^3^ to 0.94 mg/dm^3^. Interestingly, arsenate(V) ions were completely removed from the 25 and 50 mg/dm^3^ solutions in the case of X^np^-La(III) (100% sorption efficiency). The ability to remove arsenic completely at relatively low initial concentrations is very important and shows successful modification. Li et al. examined hybrid Ce-Ti sorbent in terms of arsenic removal [29]. The authors claimed that in industrial drinking water treatment, the arsenic(V) concentration usually does not exceed 0.1 mg/dm^3^ and the final arsenic concentration in wastewater must be lower than the generally accepted limit of 10 μg/dm^3^ and therefore the sorption ability to adsorb at a low initial arsenic(V) concentration is more important than the maximum sorption capacity obtained experimentally at high arsenic(V) concentration. To predict the optimal conditions of adsorbate removal both isotherm adsorption and intraparticle kinetic parameters should be determined under different system conditions because they are dependent, among others, on the concentration, temperature and interactions between the adsorbent and the adsorbate. If the adsorbent possesses particular functional groups and active centres to adsorb the adsorbate, its effect should be evaluated from the experimental data. 

Moreover, in our studies arsenic(V) ions sorption increases very sharply within the first 15 min of the phase contact time and the equilibrium appears to be reached after 120 min with the adsorption capacities equal to 4.81, 7.76, and 12.36 mg/g for X^np^ as well as 5.00, 10.00 and 16.31 mg/g for X^np^-La(III) at initial concentrations of 25, 50 and 100 mg/dm^3^, respectively. These results demonstrate that the 120-min phase contact time selected for adsorption studies is adequate for adsorption on both sorbents. The adsorption is the fastest at the beginning of the process at short time intervals and then gradually declines throughout the studied period. This indicates that as the adsorption sites on X^np^ and X^np^-La(III) surface were occupied, the rate of adsorption decreased. 

**Figure 13:**
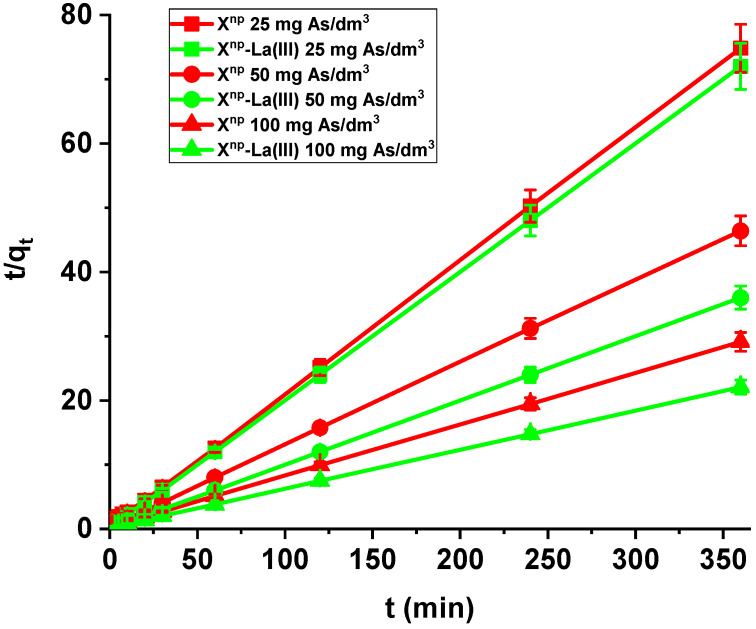
PSO plots of arsenic(V) adsorption on X^np^ (red) and X^np^-La(III) (green) (c_0_ = 25, 50, 100 mg/dm^3^, pH 6, shaking speed 180 rpm, temperature 295 K).

Comparing the kinetic studies of arsenic(V), it was found that the linear regression coefficient based on the pseudo-second order (PSO) model (*R*^2^ > 0.99) gave the best fitting (Table 4 and Table 5, Figure 13) for both X^np^ and X^np^-La(III). The As(V) adsorption rate constants determined using the PSO model are 0.030, 0.018 and 0.009 1/min, while the adsorption capacities are 3.14, 5.07, and 8.64 mg/g for X^np^. For X^np^-La(III), they are higher and equal to 0.178, 0.057, 0.020 1/min, whereas the adsorption capacities are 5.02, 10.07, and 16.46 mg/g. It is worth mentioning that the *q_e,exp_* values are well comparable with these calculated *q_e,cal_* (Table 4 and Table 5). What is more, an increase in the initial arsenic(V) concentration leads to an increase in the adsorption capacity.

It can be noticed that the adsorption involves bulk phase transport of the metal ions to the external surface of X^np^, transport across the boundary layer (external mass transfer), transport through/within X^np^ by surface diffusion and/or pore diffusion (intraparticle diffusion), and adsorption on the adsorbent surface. The last one can be affected by the stirring rate, volume, and initial concentration of the solution.

### 3.5. Equilibrium Adsorption of As(V) on X^np^-La(III)

The isotherm data were analysed using the Langmuir and Freundlich models to provide an insight into the interactions between the adsorbent and the adsorbate. Sorption equilibrium is usually described by an isotherm equation whose parameters express the affinity and surface properties of the sorbent at a fixed temperature and pH. To optimise an adsorption system, it is important to establish the most appropriate correlations for the equilibrium. Therefore, the isotherm data were analysed using the Langmuir and Freundlich isotherm models. Therefore, the isotherm data were analysed using the Langmuir and Freundlich isotherm models. The correlation parameter (*R*^2^) and the corresponding average relative errors were calculated according to the following equation:

(8) $$\Delta q\left(\%\right)=\overset{N}{\underset{i=1}{\sum }}\left|\frac{q_{exp}-q_{0}}{q_{exp}}\right|\times \frac{100}{N}$$

where: *q_exp_*—the experimental amount of arsenic adsorbed at equilib¬rium (mg/g); *q*_0_—the calculated amount of arsenic adsorbed at equilibrium and *N*—the number of the experimental data. 

**Figure 14:**
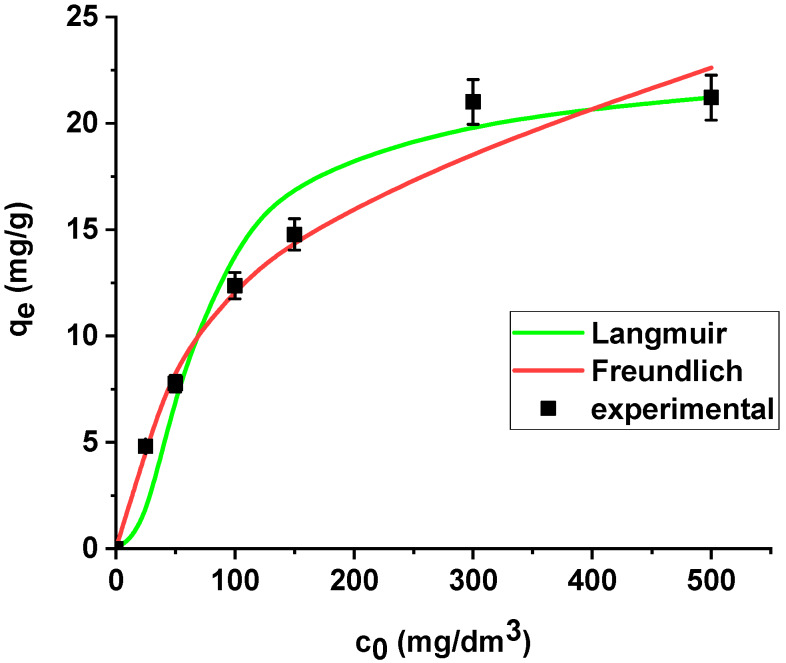
The Langmuir and Freundlich sorption isotherms of As(V) on X^np^ (pH 6, shaking speed 180 rpm, temperature 295 K).

**Figure 15:**
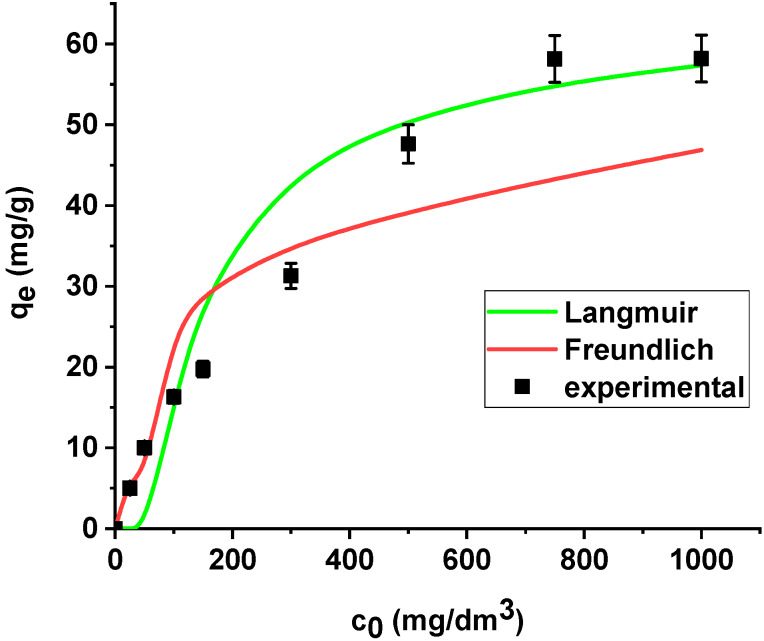
The Langmuir and Freundlich sorption isotherms of As(V) on X^np^-La(III) (pH 6, shaking speed 180 rpm, temperature 295 K).

The results are summarized in Table 6. The regression coefficients (*R*^2^) are higher for the Langmuir model in both cases, i.e., the sorption of As(V) on the unmodified and modified sorbent. What is more, the average relative errors are smaller for the Langmuir equation in both cases. The high correlation coefficients and relatively small errors suggested that the Langmuir model provides good fit of the experimental adsorption data and can be applied for description of the sorption process. The sorption capacity of X^np^ and X^np^-La(III) increased with the increase in arsenic concentration in the solution. Based on the Langmuir model, the maximum sorption capacities towards arsenic(V) were determined as 22.37 mg/g and 61.97 mg/g on X^np^ and X^np^-La(III), respectively. It is worth noting that the maximum sorption capacity of the modified sorbent is almost 3 times greater than the unmodified one. For the Freundlich model, the *K_F_* values related to arsenic(V) adsorption on the unmodified and modified ion exchanger are equal to 4.64 and 14.49, respectively (Table 6). The parameters *R_L_* (0 < *R_L_* < 1) for adsorption of arsenic(V) on both sorbents indicate that the process is favourable. The Langmuir and Freundlich sorption isotherms of As(V) on X^np^ and X^np^-La(III) are presented in Figure 14 and Figure 15.

In the case of commercial hybrid ion exchangers based on nanosized iron oxides, their quality depends on the quantity, structure, crystallinity and degree of dispersion of the iron oxides introduced, the physical form of the base polymer, the type of functional groups involved in the preparation of hybrid polymers as well as their influence on the process of adsorption [7]. The process of dispersing of iron oxide can be applied to different types of polymers such as those containing sulfonic, diphosphonic/sulfonic/carboxylic, bispicolylamine or quaternary ammonium as well as iminodiacetic functional groups:

R-SO_3_H + Fe^2+^ ⇄ R-(SO_3_)_2_Fe + 2H^+^ (9)

and slow oxidation of Fe(II) into Fe_3_O_4_ at alkaline pH, i.e.,

R-(SO_3_)_2_Fe + 2Na^+^ ⇄ 2R-SO_3_Na + Fe^2+^ (10)

as well as

3Fe^2+^ + 0.5O_2_ + 3H_2_O ⇄ Fe_3_O_4_↓ + 6H^+^ and Fe^2+^ + 2OH^−^ ⇄ Fe(OH)_2_↓ or Fe(OH)_2_↓ + 0.5O_2_ ⇄ Fe_3_O_4_↓ + 3H_2_O (11)

where: R is the resin matrix.

The magnetic properties of these sorbents are greatly influenced by the kind of functional groups. They were found to change in the following order: sulfonic > diphosphonic/sulfonic/carboxylic > bispicolylamine > iminodiacetic. However, in the case of introducing the hydrated Fe(III) oxide (HFO), to obtain hybrid ion exchangers (HIXs) the first step of preparation includes loading of Fe(III) onto the functional groups of the ion exchanger (for example sulfonic one), then desorption of Fe(III) and simultaneous precipitation of Fe(III) hydroxides within the gel and pore phase of the exchanger by passage of a solution containing both NaCl and NaOH:

R-SO_3_H + Fe^3+^ ⇄ R-(SO_3_)_3_Fe + 3H^+^ (12)

R-(SO_3_)_3_Fe + 3Na^+^ ⇄ 3R-SO_3_Na + Fe^3+^ (13)

Fe^3+^ + 3OH^−^ ⇄ Fe(OH)_3_ (14)

As for anion exchanges used as a support the following reactions can be proposed:

R-N(CH_3_)_3_Cl + FeCl_4_^−^ ⇄ R-N(CH_3_)_3_FeCl_4_ + Cl^−^ (15)

and

R-N(CH_3_)_3_FeCl_4_^−^ + OH^−^ ⇄ R-N(CH_3_)_3_ OH + Fe(OH)_3_ + Cl^−^ (16)

The next one is alcohol washing and precipitation:

Fe(OH)_3_↓ ⇄ FeO(OH) + amorphous HFO particles (17)

As for the mechanism, the adsorption of As(V) on the surface covered with iron oxides takes place in two stages. The first stage is characterized by a very high speed and consists in the diffusion of arsenic(V) to the adsorbent surface. The second stage, however, is much slower and involves the diffusion of the sorbed molecules into the micropores of iron oxides. Then, a structural transformation takes place on the adsorbent surface. In the case of arsenates(V), the process of complex formation is initially connected with the formation of monodentate and then bidentate mononuclear complexes, which transform into double-core binary complexes as the degree of polymer coverage with metal oxide increases. The speed and scale of these transformations are influenced by various factors, which include the number of active sites as well as the pH value of the solution in which the process takes place. As a result of a low pH value, the adsorption process may be affected by electrostatic interactions between the arsenate anions and the positive charge on the adsorbent surface. Under such conditions, proper ligand exchange can proceed by non-specific adsorption of arsenates(V) on the surface of iron oxides, which takes place through ion exchange with the previously adsorbed anions [30,31,32,33,34,35]. The mechanism reactions can be as follows:

R-SO_3_H/FeO(OH) + H_2_AsO_4_^−^ ⇄ [R-SO_3_H/FeO]H_2_AsO_4_ + OH^−^ (18)

2R-SO_3_H/FeO(OH) + HAsO_4_^2−^ ⇄ 2[R-SO_3_H/FeO]_2_HAsO_4_ + 2OH^−^ (19)

R-N(CH_3_)_3_(OH)/FeO(OH) + H_2_AsO_4_^−^ ⇄ [R-N(CH_3_)_3_(OH)/FeO]H_2_AsO_4_ + OH^−^ (20)

2R-N(CH_3_)_3_(OH)/FeO(OH) + HAsO_4_^2−^ ⇄ 2[R-N(CH_3_)_3_(OH)/FeO]_2_HAsO_4_ + 2OH^−^ (21)

Contrary to alkaline conditions (where only adsorption of As(V) takes place), under almost neutral conditions precipitation and adsorption can be the main mechanisms of As(V) removal. 

However, in the case of X^np^-La(III), i.e., after modification, the removal capacity was enhanced by the co-precipitation and adsorption by exchange of the OH- group with arsenic ions. At pH 6.0, the arsenic H_2_AsO_4_^−^ (about 90% in the system) and HAsO_4_^−^ (10% in the system) species are present as strong electron donors and can interact with La(III); therefore, the following reaction can be proposed:

La^3+^ + H_2_AsO_4_^−^ → LaAsO_4_ + 2H^+^ (22)

In such conditions, the insoluble lanthanum arsenate, LaAsO_4_, precipitates in the acid pH range. As for the analogous FeAsO_4_, its dissolution increases at a pH value greater than 5.0 and FeAsO_4_ can decompose at a pH greater than 6.0. Moreover, the effect of the presence of the functional groups of the ion exchanger should be neglected. The high concentration of the functional groups only allowed a high and fairly uniform loading of hydrous iron oxide particles within the polymeric matrix. Similar results were described in [36] where the removal of arsenic(V) onto lanthanum(III) modified sorbents of (hydr)oxide type was described. Additionally, it was found that with an increase of lanthanum(III) content (within Fe/La ratio from 3:1, 1:1 and 1:3), the specific surface area of the obtained composites decreased, but the pore diameter, the pore volume and their grain increased gradually (which can be explained by the increase in the particle size and shape at the highest content of La) [36]. The Langmuir adsorption capacities of 3:1, 1:1, 1:3 and 0:1 Fe–La composite (hydr)oxides were 116 mg/g, 166 mg/g, 235 mg/g, and 368 mg/g at pH 7.0, respectively. Taking into account the comparison of the examined sorbents and others used for arsenic removal (Table 7), it can be concluded that the high removal efficiency of the R-Fe–La composite (hydro)oxide makes it attractive for treatment of water contaminated with As(V). 

### 3.6. Reuse of X^np^ and X^np^-La(III)

When the equilibrium of arsenic sorption is established, the adsorbent is unserviceable and regeneration is needed to use it in a cyclic manner. The loaded adsorbent should be separated from the aqueous solution in order to perform desorption and subsequent regeneration employing an external magnetic field. The most commonly used substances desorbing arsenic ions are strong bases or strong acids, e.g., NaOH and HCl. 

**Figure 16:**
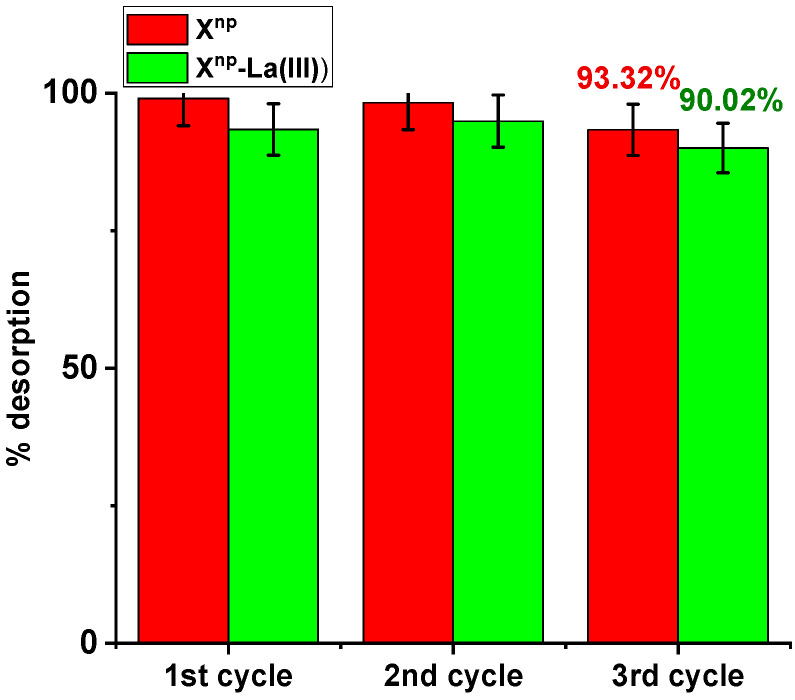
Desorption efficiency (%) for As(V) recovery using X^np^ and X^np^-La(III) (As(V) concentration: 100 mg/dm^3^, desorbing agent: 1M NaOH, sorption/desorption time: 6 h, shaking speed 180 rpm, temperature 295 K).

Three sorption and desorption cycles were carried out and the possibility of reusing the tested sorbents was assessed. Sorption was conducted using an arsenic solution of 100 mg/dm^3^ and desorption with 1M NaOH. In the case of X^np^ during the first, second and third sorption, the percentage of sorption remained relatively constant (66.67%, 66.34%, 65.34%). No significant decrease in the sorption capacity was noted. After the third cycle, the desorption effectiveness was 93.32% (Figure 16). Similar results were obtained for X^np^-La(III). The percentages of sorption amounted to 77.00%, 77.67%, and 75.85%. After the third cycle, the percentage of desorption was 90.02%. This desorption efficiency can be explained by the following mechanism:

≡FeH_2_AsO_4(s)_ + OH^−^ ⇄ ≡FeOH_(s)_ + H_2_AsO_4_^−^_(aq)_ (23)

The obtained results clearly indicate that the arsenic adsorption can be carried out repeatedly on the same material after NaOH regeneration. The ability to reuse the same material significantly reduces costs. 

1 M NaOH was used for the regeneration of maghemite and it was found that after six cycles the adsorbent maintained over 40% of the initial sorption capacity [45]. In turn, arsenic adsorbed on granular iron and cerium hydroxide (GFC) was subjected to elution of 1 M NaOH and the recovery efficiency was equal to 89%. After that, As(V) was removed with the same efficiency [46]. Another example could be the regeneration of Fe-Cu double oxide with NaOH. After 4 cycles of adsorption and desorption, the sorption capacity decreased by only 6.2% regarding As(V) [47]. Also the spent Fe–La composite (hydr)oxide could be effectively regenerated using NaOH solution and reused several times [36].

## 4. Conclusions

The paper has proved that earlier adsorption of lanthanum(III) ions on the iron oxide ion exchanger contributed to an increase in the sorption efficiency of arsenic(V) ions.

(a) The maximum sorption capacity for arsenic ions was almost 3 times greater after the modification.
(b) X^np^-La(III) removed arsenic entirely from the solution of 50 mg/dm^3^ in a relatively short time (about 2 h).
(c) It was found that after modification the sorbent can be successfully reused for purification of water contaminated with arsenic. After 3 cycles of adsorption and desorption, no significant decrease in the process efficiency was observed.
(d) Under almost neutral conditions precipitation and adsorption can be the main mechanisms of As(V) removal. After modification, the removal capacity was enhanced by the co-precipitation and adsorption by exchange of the OH− group with arsenic ions.

The threefold greater maximum sorption capacity after the modification together with the short duration time of the process lead to a reduction in operating costs. What is more, the arsenic removal process is very efficient at the pH of municipal waters. A lack of significant decrease in the process efficiency, even after 3 cycles of adsorption and desorption, enables the multiple use of the same material. All these factors confirmed that lanthanum-modified X^np^ achieves better results than unmodified X^np^ and is a very promising material in the context of arsenic removal from contaminated water bodies. The modification process itself is a great opportunity to improve the properties of iron oxide containing sorbents and to achieve the WHO restrictive limit for arsenic. 

## Figures and Tables

**Table 1:** Properties of Purolite Arsen X^np^.

Polymer Structure	Divinylbenzene Crosslinked with Polystyrene
Matrix structure	Macroporous
Physical form and appearance	Reddish-brown spherical particles
Particle size range	0.300–1.200 mm
Maximum working temperature	80 °C
Working pH range	4–9

**Table 2:** Physical properties of X^np^ and X^np^-La(III).

Ion Exchanger	X^np^	X^np^-La(III)
Specific surface area (*S_BET_*) (m^2^/g)	55.27	52.75
Total pore volume (*V_t_*) (cm^3^/g)	0.189	0.169
Average pore diameter (*D_p_*) (nm)	13.70	12.79

**Table 3:** Kinetic parameters of lanthanum(III) adsorption on X^np^.

Kinetic Parameters	X^np^-La(III)		
	10 (mg/dm^3^)	50 (mg/dm^3^)	100 (mg/dm^3^)
**PFO**			
***q*_1,*cal*_**	0.01	2.06	6.71
***k*_1_**	0.025	0.016	0.011
***R*^2^**	0.6537	0.6786	0.9105
**PSO**			
***q*_2,*cal*_**	2.00	9.89	17.87
***k*_2_**	8.968	0.045	0.010
***h***	35.843	4.406	3.131
***R*^2^**	1.0000	0.9999	0.9986

Where: *q*_1,*cal*_ and *q*_2,*cal*_—the calculated amount of arsenic adsorbed at equilibrium for the PFO and PSO models; *k*_1_ and *k*_2_ are the reaction rate constants of the pseudo-first order (1/min) and pseudo-second order (g/mg min), *R*^2^—the correlation coefficient; *h*—the initial rate of adsorption for the PSO model (mg/g min).

**Table 4:** Kinetic parameters of arsenic(V) adsorption on X^np^.

Kinetic Parameters	X^np^-As(V)		
	25 (mg/dm^3^)	50 (mg/dm^3^)	100 (mg/dm^3^)
**PFO**			
***q*_1,*cal*_**	0.83	1.70	4.55
***k*_1_**	0.032	0.031	0.032
***R*^2^**	0.4355	0.5807	0.7847
**PSO**			
***q*_2,*cal*_**	3.14	5.07	8.64
***k*_2_**	0.030	0.018	0.009
***h***	0.292	0.460	0.702
***R*^2^**	0.9999	0.9999	0.9999

**Table 5:** Kinetic parameters of arsenic(V) adsorption on X^np^-La(III).

Kinetic Parameters	X^np^-As(V)-La(III)		
	25 (mg/dm^3^)	50 (mg/dm^3^)	100 (mg/dm^3^)
**PFO**			
***q*_1,*cal*_**	0.29	1.34	5.09
***k*_1_**	0.029	0.036	0.021
***R*^2^**	0.4854	0.6614	0.8920
**PSO**			
***q*_2,*cal*_**	5.02	10.07	16.46
***k*_2_**	0.178	0.057	0.020
***h***	4.488	5.825	5.306
***R*^2^**	0.9999	0.9999	1.0000

**Table 6:** The Langmuir and Freundlich parameters for adsorption of arsenic(V) on X^np^ and X^np^-La(III) (pH 6, shaking speed 180 rpm, temperature 295 K).

Isotherm Parameters	X^np^-As(V)	X^np^-La(III)-As(V)
**Langmuir Model**		
***q*_0_**	22.37	61.97
***K_L_***	0.047	0.017
***R_L_***	0.462	0.696
***R*^2^**	0.9931	0.9637
***error (%)***	5.47	6.54
**Freundlich Model**		
***K_F_***	4.64	14.49
***n***	3.775	5.589
***R*^2^**	0.9790	0.8611
***error (%)***	6.50	18.28

**Table 7:** The comparison of other sorbents used for arsenic(V) removal.

Adsorbent Type	pH	As(V) (mg/g)	Ref.
La and Ce-loaded orange waste gels	6.0–9.5	42	[37]
La-modified ceramic material	4.0–8.0	23	[38]
Ce oxide modified activated carbon	5.0	43.6	[39]
Fe–La composite (hydr)oxide	7.0	235	[36]
Fe_3_O_4_@SiO_2_@TiO_2_ nanosorbent	9.0	10.2	[40]
Mg doped α-Fe_2_O_3_	7.0	10	[41]
Fe_3_O_4_	8.2	12.56	[42]
Nanoscale zero-valent iron-reduce graphite oxide modified composite	7.0	29.04	[43]
Hydrated ferric hydroxide	9.0	7.0	[44]
X^np^	6.0	22.37	this study
X^np^-La(III)	6.0	61.97	this study
